# What are the Effects of Exercise on Trabecular Microarchitecture in Older Adults? A Systematic Review and Meta-analysis of HR-pQCT Studies

**DOI:** 10.1007/s00223-023-01127-7

**Published:** 2023-09-19

**Authors:** Thomas Z. Schlacht, Inaya Haque, Dawn A. Skelton

**Affiliations:** Research Centre for Health (ReaCH), Physiotherapy and Paramedicine, Glasgow Caledonian University, Cowcaddens Road, Glasgow, G4 0BA UK

**Keywords:** Trabecular microarchitecture, High-resolution peripheral quantitative computed tomography, Bone quality, Exercise, Older adults, Review

## Abstract

The objective of this review was to determine the effects of exercise on high-resolution peripheral quantitative computed tomography (HR-pQCT) derived trabecular microarchitecture parameters in older adults. Five electronic databases were systematically searched by two independent reviewers. Inclusion criteria were adults age ≥ 50, any type of exercise as part of the intervention, and trabecular microarchitecture assessed via HR-pQCT. Data was extracted from included studies, and where suitable, included in a meta-analysis. Quality of included studies was appraised. Seven studies (397 participants) were included. All participants were postmenopausal women. Interventions included jumping, whole-body vibration, and power/plyometric training. All studies were rated as either weak or moderate quality. Meta-analysis (5 studies) showed no significant changes in any parameters when considering all exercise or sub-analysing based on type. Exercise was not found to have significant effects on trabecular microarchitecture in postmenopausal women over the age of 50. These findings should be interpreted with caution due to the small number of studies investigating few modes of exercise, their weak to moderate quality, and risk of bias. High-quality studies are needed to determine the effects of additional types of exercise in a more diverse population of older adults, including men.

**Supplementary Information:**

The online version contains supplementary material available at 10.1007/s00223-023-01127-7.

## Introduction

Decreases in short- and long-term health-related quality of life [1] and increased risk of mortality [2] are common after older adults sustain a fracture. Once men and women reach the age of 50, their chances of sustaining a future fracture are 20% and 50%, respectively [3]. Exercise and physical activity (PA) are often promoted as effective strategies to increase bone strength and decrease fracture risk [4, 5]. It is important, however, to distinguish between these terms when studying their effects on bone morphology because they may elicit different outcomes [6]. PA is any movement of the body that is generated by skeletal muscles requiring energy expenditure, whereas exercise is a subgroup of PA that is structured, planned, repetitive, and has the goal of improving or preserving physical fitness [7].

Exercise has been found to reduce fracture risk in older adults [8], although its underlying mechanisms are not entirely understood. One of the ways it contributes is by reducing fall risk, frequency, and severity [9, 10]. Physiologically, increased bone mineral density (BMD) is associated with reductions in fracture risk [11], however it has been suggested that only 60–70% of the variance in bone strength can be attributed to BMD [12, 13]. While research supports the beneficial effects of certain types of exercise such as resistance training and impact activities on BMD [14, 15], activities such as swimming may help to strengthen bones without increasing BMD [16]. Furthermore, over half of the non-vertebral fractures in adults over the age of 55 occur in those who, based on their BMD, do not have osteoporosis [17]. It is therefore evident that other variables contribute to bone strength.

Bone quality refers to components of bone structure and composition other than BMD that influence bone strength [18]. It encompasses elements such as cortical microarchitecture, trabecular microarchitecture (TbM), bone turnover, mineralisation, and its matrix and mineral composition [18]. Although research has investigated the effects of exercise on BMD, few studies have examined its effect on bone quality.

TbM is a key element of bone quality [19] that refers to the level of organisation of individual trabeculae (plate and rod-like structures that are arranged in a honeycomb pattern) [20], and generally encompasses trabecular bone volume fraction (BV/TV), number (TbN), thickness (TbTh), and separation (TbSp) [19, 21]. BV/TV is the ratio of trabecular bone volume to total volume, TbN represents the average number of trabeculae per millimeter, TbTh is the average width of each trabecula, and TbSp is the average distance separating trabeculae [19, 21]. Although only 20% of the human skeleton is composed of trabecular bone [22], certain anatomical regions with high amounts of trabecular bone such as the vertebrae, proximal femur, and distal radius are common sites of fragility fractures [23, 24]. Furthermore, due to the greater metabolic activity in trabecular bone compared to cortical bone, the former is usually affected first by osteoporotic bone loss [25]. Age-related degradation typically presents as decreased BV/TV, TbN, and TbTh and increased TbSp, although differences exist between sexes and anatomical regions [26]. Moreover, TbN and TbTh are important factors in the prediction of fractures in older adults [25, 27]. It is therefore critical to develop strategies that help minimise the negative impact of ageing on TbM.

High-resolution peripheral quantitative computed tomography (HR-pQCT) is the instrument of choice to measure bone microarchitecture in humans [28]. In addition to its standardised protocols in adults [28, 29], HR-pQCT has demonstrated reproducibility [29, 30] and has been validated against the gold standard micro-computed tomography [31, 32], which has limitations for clinical use in vivo in humans [33]. While other bone imaging tools such as quantitative computed tomography (QCT), the original peripheral QCT, and magnetic resonance imaging can differentiate between cortical and trabecular bone, they each face limitations in their resolution, accuracy, or standardisation [28]. This renders them suboptimal for TbM evaluation. Although HR-pQCT can only scan the distal limbs, these areas are high in trabecular bone and fractures are seen at these sites, especially in older osteoporotic women [23, 24]. While currently sparse in clinical settings [34], HR-pQCT is becoming more common with normative data emerging for various populations [35, 36].

Limited research has examined the effects of exercise on TbM, although two reviews have investigated its effect on standard peripheral QCT parameters in postmenopausal women. Hamilton et al. [37] and Polidoulis et al. [38] conducted similar reviews but disagreed on the extent to which exercise affected trabecular bone. As multiple studies were included in both reviews, their contradicting inferences may indicate a discrepancy in the interpretation of the small body of evidence. Furthermore, as none of the studies in either review used HR-pQCT, changes in TbM were not examined, leaving an important research gap unfilled. As a decade has passed since the publication of both reviews, studies examining the effects of exercise on TbM have likely been conducted. These limitations and contradictory findings validate the need for further research.

Understanding the effects of exercise on bone quality in older adults would be valuable information to help inform exercise guidelines and prevent fragility fractures in this population. The aim of this study is therefore to systematically review and evaluate the evidence on the effects of exercise on HR-pQCT derived TbM parameters in adults aged over 50.

## Methods

This systematic review was conducted following the Preferred Reporting Items for Systematic Reviews and Meta-Analyses 2020 reporting format [39].

### Information Sources and Search Strategy

The literature search was conducted on March 18^th^ 2022, in five electronic databases: MEDLINE, CINAHL, Web of Science, PEDro, and Cochrane CENTRAL. Reference lists of included articles were also scanned for studies which may have been missed by the electronic search. Searches were constructed using a combination of keywords, subject headings, and Medical Subject Headings related to the population (adults aged 50 and over), intervention (exercise), and outcome (TbM parameters measured using HR-pQCT). Due to the variable terminology used to refer to TbM, as well as the frequent interchanging use of the terms *physical activity* and *exercise*, a broad search strategy was used to minimise the risk of missing relevant studies. The full search strategy can be found in Online Resource 1.

All articles retrieved were exported to Rayyan Intelligent Systematic Review [40] where duplicates were removed by the first author (TS). Two independent reviewers (TS and IH) then screened results by title and abstract. Once agreement was achieved, full texts were retrieved. The two independent reviewers then assessed texts using the established eligibility criteria. Again, discrepancies were resolved through discussion. A third independent reviewer (DS) was available for consultation.

### Eligibility Criteria

Studies examining the effect of exercise on HR-pQCT derived TbM parameters were included in this review. Inclusion criteria were 1) average age of participants 50 years or over, 2) any mode of exercise as part of the intervention, 3) BV/TV, TbN, TbTh, and/or TbSp measured using HR-pQCT, 4) full text available in English, and 5) peer-reviewed studies. Participant health status did not contribute to eligibility criteria. It was anticipated that few studies would be found, therefore limited exclusion criteria were applied. Exclusion criteria were 1) narrative reviews, systematic reviews, meta-analyses, study proposals, protocol papers, and abstracts, 2) TbM measured using devices other than HR-pQCT, 3) language other than English, 4) animal trials, and 5) cadaveric studies.

### Data Extraction and Analysis

The first author (TS) extracted data into Microsoft Excel version 16.58. To standardise parameter units, in cases where BV/TV and TbTh were reported in decimal points and micrometres, they were converted to percentages and millimetres, respectively. Approximately 40% of extracted data was checked by an independent reviewer (IH).

Outcomes were measures of BV/TV, TbN, TbTh, and TbSp, taken at the distal tibia and/or radius. HR-pQCT was chosen to assess these parameters because it is the recommended bone imaging device for bone microarchitectural examination in humans [28], and standardisation of the assessment tool facilitated comparison between studies.

Post-intervention means and standard deviations were inputted into Review Manager (RevMan) version 5.4 [41] to conduct a meta-analysis. In cases where studies reported confidence intervals (CI), they were converted to standard deviations [42]. Randomised controlled trials (RCTs) which reported follow-up measurements were included; those which reported only mean absolute change or lacked a control group were not. For studies that took scans at multiple timepoints, only the measurements immediately succeeding the intervention period were inputted. All outcomes were continuous. The inverse variance method, fixed effect model, and mean difference were used. Analysis details included totals and subtotals, along with 95% study and total CIs. Heterogeneity was assessed using the Chi^2^ and I^2^ statistics alongside the accompanying p-value. Outcomes of 0–40% were considered low, 30–60% moderate, 50–90% substantial, and 75–100% high [42].

### Quality Appraisal

The Effective Public Health Practice Project (EPHPP) Quality Assessment Tool for Quantitative Studies was used by TS to evaluate the quality and risk of bias of the included studies [43]. Approximately 40% of the quality assessment was also completed by a second reviewer (IH), with any discrepancies resolved through discussion. This validated tool assesses the following six components of a study to assign it a global rating of weak, moderate, or strong: selection bias, study design, confounders, blinding, data collection methods, and withdrawals and dropouts [43]. The EPHPP global ratings were used to ascribe credibility to study conclusions. Weak, moderate, and strong scores are resultant of low, medium, and high study quality. Credibility was given to study conclusions accordingly.

## Results

### Study Selection

**Fig. 1:**
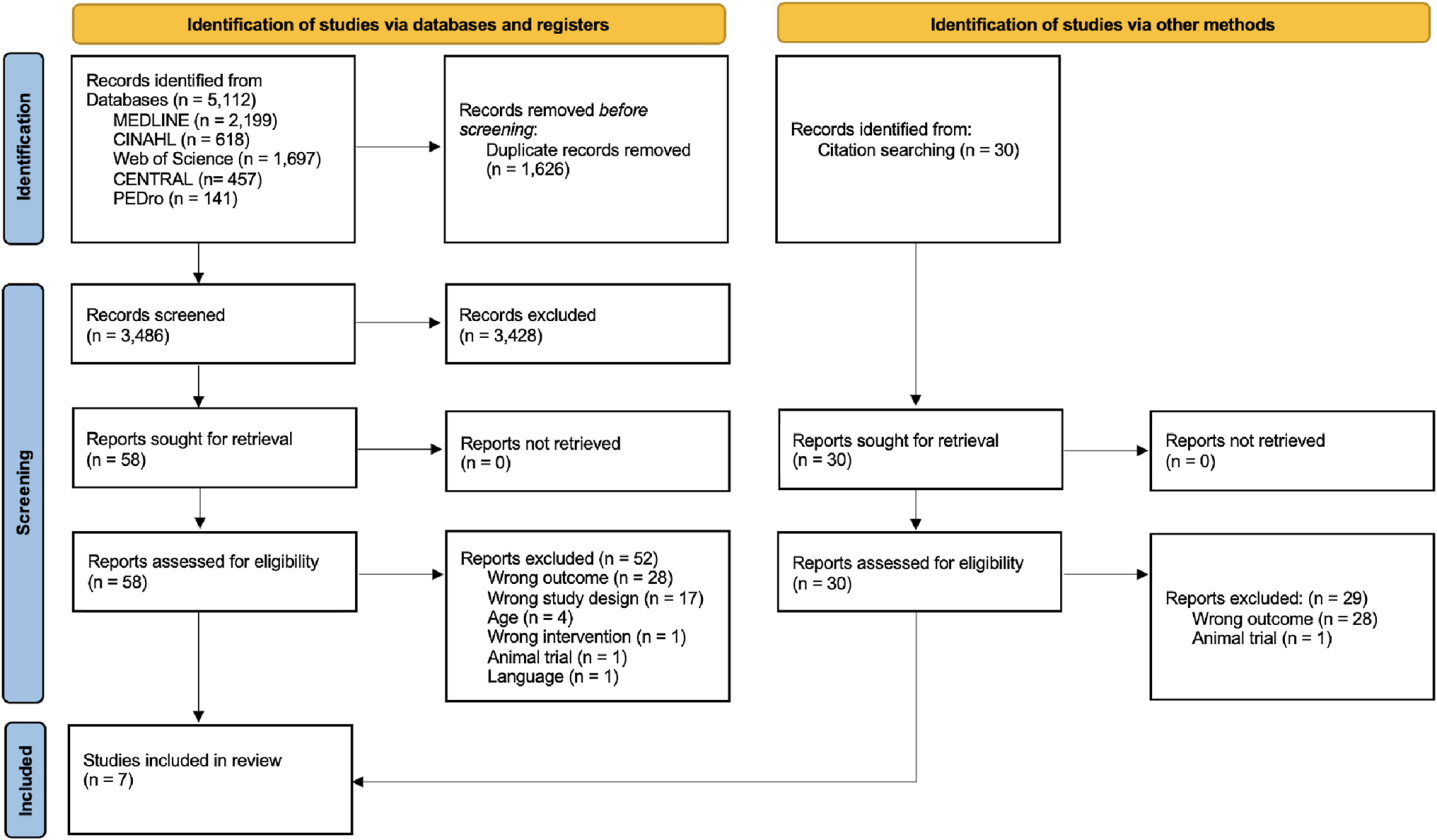
PRISMA flow chart illustrating the study selection process. Key: MEDLINE = Medical Literature Analysis and Retrieval System Online, CINAHL = Cumulated Index to Nursing and Allied Health Literature, CENTRAL = Cochrane Central Register of Controlled Trials, PEDro = Physiotherapy Evidence Database

The initial search strategy identified 5,112 articles from the selected databases (Fig. 1). After removing 1,626 duplicates, 3,486 records remained to be screened by title and abstract. During this process, 3,428 were removed. Of the remaining 58 requiring a full-text screening, 52 were excluded based on eligibility criteria. The most common reasons were that they assessed the wrong outcome or were the wrong study design. The remaining six studies were included in this review [44–49]. While searching their reference lists for relevant articles that were not discovered via the database search, one additional study was found [50] (Fig. 1).

### Study Characteristics

A summary of study characteristics can be found in Table 1. Studies were conducted in six countries: Australia [47], Brazil [48], Canada [46, 50], Denmark [45], Sweden [49], and the UK [44]. Five studies [44–46, 48, 50] were RCTs, one [47] was a non-randomised pilot study with single-arm pre- and post-measurements, and one [49] was a non-randomised intervention study. Of the six studies [44–46, 48–50] that used control groups, two [44, 49] used single participants as both the intervention and control, with one leg as the exercise leg and the other as the control leg. One study [44] did not use participant randomisation and instead randomised the selection of the intervention leg within participants.

**Table 1:** Included studies—study and participant characteristics

First author, year, country	Study design	Aim	Number of participants (N_1_/N_2_), age, sex	Eligibility criteria	Study setting and supervision	Adherence assessment method and outcome	Author's conclusions
Du, 2021 [44], United Kingdom	Randomised controlled trial	Primary: Investigate the effects of a high-impact exercise programme on global trabecular bone volume in healthy postmenopausal women Secondary: Examine localised changes in TbM and localised bone remodelling rates in the exercise leg compared to the control leg	IG* = 10/9 CG* = 10/9 Age (all participants): 63 ± 4 Women	Inclusion: women aged 55–70 years, at least 12 months post-menopause, BMI < 30 kg/m^2^, no diagnosis of osteoporosis or knee osteoarthritis, no other contraindications to exercise Exclusion: completed > 1 bout of high-impact exercise/week (with likely ground reaction force greater than jogging) or taking medication known to affect bone metabolism	Participant's home, unsupervised	Diary 80.4 ± 24.0% (mean ± SD) during the final 14 weeks (full protocol)	A 6-month high-impact exercise programme can increase TbN and may improve BV/TV of the distal tibia
Jepsen, 2019 [45], Denmark	Randomised controlled trial	Investigate the effect of combining WBV and teriparatide on BMD, bone microarchitecture, and bone turnover markers compared to teriparatide alone in postmenopausal women with severe osteoporosis	IG = 17/15 CG = 18/18 Age: IG = 69 ± 5 CG = 69 ± 8 Women	Inclusion: postmenopausal women ≥ 50 years of age with either one vertebral fracture within the last 3 years with > 25% reduction in vertebral height and T-score < − 3 at the lumbar spine or total hip, or at least two vertebral fractures with > 25% reduction in vertebral height with no additional requirements for low BMD, planned to start treatment with teriparatide Exclusion: ongoing oral glucocorticoid treatment, inability to tolerate WBV for 1 min at screening, or contraindications to WBV, such as the presence of pacemakers or joint replacements	Participant's home, unsupervised	WBV = self-reported in a logbook Teriparatide = looked in prescription database WBV adherence was > 75% in 13 participants (76%) Teriparatide adherence was > 80% in 33 participants (94%) based on the number of collected prescriptions (of planned 13 a year)	No effect on bone microarchitecture was found in any groups
Liphardt, 2015 [46], Canada	Randomised controlled trial	Monitor changes in bone microarchitecture and bone strength of the distal radius and tibia in osteopenic postmenopausal women participating in a 12-month exercise programme based on WBV training	IG = 22/17 CG = 20/14 Age: IG = 59.1 ± 4.6 CG = 58.5 ± 3.3 Women	Inclusion: postmenopausal and a DXA T-score of the femoral neck or lumbar spine in the osteopenic range (− 1.0 to − 2.5). Postmenopausal was defined as > 5 years since the last menses	Bone imaging laboratory, supervised	Supervision 90% compliance (119 out of 132 sessions)	WBV training did not lead to measurable improved bone quality in osteopenic postmenopausal women after 12 months of training compared to the CG
Ng, 2021 [47], Australia	Non-randomised pilot with single-arm pre- and post-test	Primary: Determine the feasibility and safety of a 16-week impact exercise programme performed primarily in the home, and to explore factors associated with adherence rates Secondary: Investigate the effectiveness of the programme on hip and spine BMD, bone microarchitecture, physical function, and bone turnover markers	IG = 50/44 No CG Age: 64.5 ± 7.5 Women	Inclusion: BMD T-score < − 1.0 at the lumbar spine, total hip or femoral neck confirmed by DXA, either performed by the study investigators or verified by a physician in the past 2 years, BMI < 30 kg/m^2^, participation in < 150 min of self-reported moderate to vigorous physical activity per week Exclusion: surgery within 6 months, knee or hip osteoarthritis, severely impaired physical function that would limit exercise participation, regular corticosteroid use within 3 months, or any progressive neurological disorder	Participant's home, unsupervised An individual supervised session in the medical centre was held at baseline, week 5, and week 9	Exercise diary Supervised exercise visits = 100% adherence Home-based exercises = 85.3 ± 17.3% (73 ± 15 out of the prescribed 85 sessions) 15.9% (n = 7) of participants had 100% adherence to the exercise program 77.3% (n = 34) had a high adherence rate of ≥ 80% and only one participant had an adherence of < 50%	There were no significant changes in TbM
Pinho, 2020 [48], Brazil	Randomised controlled trial	Investigate the effects of a 20-week power and plyometric training programme on lumbar spine and tibia bone microstructure and function. Additionally, to determine if this intervention time is enough to induce measurable changes in bone structure and function	IG = 21/21 CG = 17/17 Age (mean and 95% CI): IG = 66.9 (65.0–68.8) CG = 65 (63.3–66.7) Women	Inclusion: women aged 60–70 years, absence of cardiovascular, osteoarticular, musculoskeletal or neurological disorders, uncompensated visual problems, depression or mental illness, negative history of falling or dizziness during one year prior to the study, absence of osteometabolic diseases or chronic diseases, and not using medication that may interfere with bone metabolism Exclusion: attending less than 75% of the exercise sessions (for the IG) and the absence in the final evaluation (both groups)	Unclear setting, supervised with 1:2 instructor to participant ratio	Participants in the IG attended on average 92% of the planned training sessions. None attended less than 80%	The 20-week power/plyometric training protocol was able to improve tibial bone age-related microstructure degradation
Sundh, 2018 [49], Sweden	Non-randomised intervention study	Investigate the effect of a 3-month unilateral high-impact exercise program on bone material properties and microarchitecture in healthy postmenopausal women	IG* = 20/20 CG* = 20/20 Age (all participants): 55.5 ± 2.3 Women	Inclusion: healthy postmenopausal women age 50–60 Exclusion: history of osteoporosis, regular weight-loading exercise (> 1 time per week the last 3 months), still menstruating, current smoking, current or past (within 6 months) hormone-replacement therapy, fracture located at the ankle or lower leg, diseases or use of medication known to influence bone metabolism or fracture risk, and those who had initiated calcium or vitamin D supplementation in the preceding 6 months	Participant's home, unsupervised	Calculated by summing the reported number of days with performed jumps divided by the total number of days for the intervention Overall compliance to the exercise program was 97.8% (IQR 90.8% to 100%)	No changes were observed for bone geometry and microarchitecture traits
Slatkovska, 2011 [50], Canada	Randomised controlled trial	Examine the effect of WBV on volumetric BMD and bone structure at the distal tibia and radius and areal BMD at the hip and spine in postmenopausal women who received calcium and vitamin D supplementation	IG 1—90 Hz WBV = 67/65 IG 2—30 Hz WBV = 68/65 CG = 67/65 (all baseline participants were included in intention to treat analysis) Age: IG 1 = 60.5 ± 7 IG 2 = 59.6 ± 6 CG = 60.8 ± 5.5 Women	Inclusion: experienced menopause 1 ≤ years ago and their lowest BMD T-score at the lumbar spine, femoral neck, or total hip was between -1.0 and -2.5 Exclusion: BMD T-score > -1.0 or ≤ -2.5, fragility fracture after age 40, secondary causes of bone loss, metabolic bone diseases, diseases affecting bone metabolism, active cancer in the past 5 years, body mass ≥ 90 kg, knee or hip joint replacements, spinal implants, hormone therapy in the past 12 months, bisphosphonates in the past 3 months or ever for ≥ 3 months, raloxifene or teriparatide in the past 6 months, long-term glucocorticoid, anticoagulant, or anticonvulsant therapy, could not tolerate WBV for 20 min at screening, expected changes in physical activity levels, or expected to travel for > 4 consecutive weeks during the study	Participant's home, unsupervised	Self-reported at 6 months (for feedback only) and extracted platforms at 12 months using an internal digital clock recording of the date, time, and duration of every session Median estimate of adherence (IQR): Cumulative duration IG 1 = 79% (41–91%) IG 2 = 77% (55–86%) Number of days IG 1 = 70% (33–82%) IG 2 = 65% (52–80%) Full session count IG 1 = 78% (41–91%) IG 2 = 77% (53–86%) Median self-reported adherence to calcium and vitamin D supplementation (mean): IG 1 = 98% (90%) IG 2 = 98% (89%) CG = 96% (89%)	12 months of low-magnitude (0.3* g*) WBV at either 90 or 30 Hz had no effect on BMD or bone structure in healthy, community-dwelling, postmenopausal women who received calcium and vitamin D supplementation

Age is denoted as mean ± standard deviation at baseline unless otherwise stated

*N*_*1*_  number of participants at baseline, *N*_*2*_ number of participants at follow-up, *TbM* trabecular microarchitecture, *IG* intervention group, *CG* control group, *BMI* body mass index, *SD* standard deviation, *TbN* trabecular number, *BV/TV* trabecular bone volume fraction, *WBV* whole-body vibration, *BMD *bone mineral density, *DXA* dual-energy x-ray absorptiometry, *CI* confidence interval, *IQR* interquartile range. IG* represents the intervention leg and CG* represents the control leg of single participants

### Baseline Participant Characteristics

Details of participant characteristics can be found in Table 1. The seven studies included a total of 397 participants, all of whom were post-menopausal women. Participants per study ranged from 10 [44] to 202 [50]. Three studies [44, 48, 49] excluded individuals with osteoporosis, while all participants in one study [45] were osteoporotic. One study [47] included women with either osteoporosis or osteopenia, and two studies [46, 50] required participants to be osteopenic. Participants of one study [44] were a subgroup of a larger study [51] that volunteered to undergo HR-pQCT scans.

### Intervention

Intervention details are presented in Table 2. Exercise interventions ranged from 3 [49] to 12 [45, 46, 50] months. Three studies [44, 47, 49] used jumping as their intervention, three [45, 46, 50] used whole body vibration (WBV), and one [48] used a power and plyometric training programme. Participants in all three jumping studies performed the jumps on one leg, however two of the studies [44, 49] used the opposite leg as a control with no jumps being performed on it, whereas the third [47] performed the single-leg jumps on both legs. WBV was considered exercise because users’ muscles work to absorb the oscillatory forces [7, 52]. Each of the WBV studies used different machine parameters. Jepsen et al. [45] administered teriparatide treatment to participants in both the intervention and control groups, whereas participants in five studies [44, 47–50] were not taking medications for osteoporosis or that are known to influence bone metabolism, however, Slatkovska et al. [50] gave all participants calcium and vitamin D supplements. Liphardt et al. [46] inquired about medication use but did not report findings.

**Table 2:** Included studies—intervention details

First author, year	Intervention	Control	Intervention protocol	Exercise frequency	Intervention length	Progression	Notes
Du, 2021 [44]	Unilateral jumping programme on an exercise leg	Non-exercise leg	After a five-minute warm-up [51], hops were completed in 3–5 sets with 15 s of rest in between	Daily	6 months	Participants were individually progressed for the first 10 weeks to a final protocol of 50 multidirectional hops per day from week 11–26 Hops were vertical in direction until week 6, at which point anterior/posterior, lateral, and rotational hops were introduced [51] Participants were encouraged to increase their jump height over time [51]	Participants were advised to complete the protocol in shoes they would wear for exercise and on a firm surface [51]
Jepsen, 2019 [45]	WBV training with subcutaneous teriparatide treatment (20 μg/day)	Subcutaneous teriparatide treatment (20 μg/day) without WBV training	12 min of training with a ratio of 1:1 WBV to rest ratio (6 min of vibration) WBV was conducted at a frequency of 30 Hz, an amplitude of 1 mm (low displacement), and peak acceleration of 35.53 ms^−2^ root-mean-square (3.6 g) WBV was performed with the knees bent at approximately 20 degrees	3 days per week with a day off in between	12 months	Training gradually progressed up to 6 min of WBV time during the first eight weeks	N/A
Liphardt, 2015 [46]	WBV training	No intervention and advised not to change the daily routines regarding nutrition and physical activity	Amplitude of 3–4 mm at 20 Hz was applied for 10 min per training day Subjects trained in a stable position at 30 degrees of knee flexion WBV was completed in 10 sets of 1-min with vibration and a 1-min break between vibration bouts	2–3 sessions per week (11 sessions per month)	12 months	N/A	N/A
Ng, 2021 [47]	Unilateral jumping programme on both legs	No control group	Each exercise session consisted of a brief warm-up (slow walking and stretching) followed by unilateral hopping on each leg in sets of 10	Week 1–4: 3 days per week Week 5–8: 5 days per week Week 9–16: 7 days per week	16 weeks	Week 1–4: 3 sets of 10 hops per leg, low to medium height, jumping vertically Week 5–8: 4 sets of 10 hops per leg, medium to high height, jumping forwards Week 9–16: 5 sets of 10 hops per leg, high height, jumping sideways	Participants who were initially not confident in their balance were advised to hold onto a fixed support while hopping from weeks 1 to 4
Pinho, 2020 [48]	Power and plyometric exercise programme	No exercise programme	Training sessions were 60 min Sessions were divided into a main part (55 min) and a cool down with stretching exercises. 14 exercise stations were included: drop jump (two stations), squat jump, leg press, knee extension, knee flexion, ankle dorsiflexion in a low pulley, body weight ankle plantarflexion, chest press, seated row, abdominal muscles exercise and three rest stations. Three sets of 10 repetitions were performed at each station. Every three exercises, a resting station was given	3 non-consecutive days per week	20 weeks	The first two weeks were used as a familiarization period with the training. At week three, and every four weeks thereafter, estimation of 1RM was obtained for each exercise and the load intensity for lower (50% of 1RM) and upper limbs (60% of 1RM) were established	Station order was changed each session. Jump stations were distributed over the training session For the drop jump exercise, a step with 9 cm height was used (for the first six weeks) that later was replaced by a step with 18 cm height
Sundh, 2018 [49]	Unilateral jumping programme on an exercise leg	Non-exercise leg	Participants performed a specific number of hops each day	Daily	3 months	Week 1: 3 sets of 10 Week 2: 3 sets of 15 Weeks 3 to 6: 3 sets of 20 Weeks 7 to 12: 4 sets of 20	The one-legged jumps were performed without shoes Participants were instructed to jump and land on the back of the foot without mitigating the shock
Slatkovska, 2011 [50]	WBV training with calcium and vitamin D supplementation	Calcium and vitamin D supplementation without WBV	Participants were given a synchronous 0.3 g-WBV platform that oscillated at either 90 or 30 Hz (peak-to-peak displacement, < 50 µm) Participants were instructed to stand on platform for 20 min per day with erect, neutral posture at the neck, lumbar spine, and knees, wearing socks or barefoot, and without excessive foot or body movements	Daily	12 months	N/A	Participants were provided with calcium and vitamin D supplements so that their daily intake from diet plus supplements approximated 1200 mg and 1000 IU, respectively

*WBV* whole-body vibration, *1RM* 1 repetition maximum

### Outcome

Details of each study’s results can be found in Table 3. Six studies [44–46, 48–50] used the XtremeCT HR-pQCT device, whereas one [47] used the newer XtremeCT II. Five studies [44, 46–48, 50] measured all four TbM parameters, while two [45, 49] did not measure TbSp. All seven studies took measurements at the same location of the distal tibia, and each of the WBV studies also scanned a common site at the distal radius. All studies except for one [48] reportedly followed previously standardised scanning protocols.

**Table 3:** Included studies—results

First author, year	HR-pQCT model	Scan location	Intervention group baseline measurements	Intervention group intermediate measurement	Intervention group follow-up measurements	Control group baseline measurements	Control group intermediate measurements	Control group follow-up measurements
Du, 2021 [44]	XtremeCT, Scanco Medical	Distal tibia of both legs	BV/TV: 12.6 ± 1.5* TbN: 1.698 ± 0.131 TbTh: 0.074 ± 0.011 TbSp: 0.519 ± 0.047	N/A	6 months BV/TV: 12.7 ± 2.0* TbN: 1.779 ± 0.201 TbTh: 0.071 ± 0.014 TbSp: 0.498 ± 0.068	BV/TV: 12.6 ± 2.0* TbN: 1.768 ± 0.184 TbTh: 0.071 ± 0.012 TbSp: 0.500 ± 0.055	N/A	6 months BV/TV: 12.6 ± 1.7* TbN: 1.753 ± 0.180 TbTh: 0.072 ± 0.005 TbSp: 0.504 ± 0.060
Jepsen, 2019 [45]	XtremeCT, Scanco Medical	Non-dominant distal radius and tibia. The dominant limb was scanned when participants had previous fractures at the desired site	*Distal tibia* BV/TV: 9.33 ± 2.95 TbN: 1.42 ± 0.50 TbTh: 0.068 ± 0.016 *Distal radius* BV/TV: 7.68 ± 2.88 TbN: 1.38 ± 0.37 TbTh: 0.056 ± 0.012	6 months *Distal tibia* BV/TV: 9.15 ± 4.01 TbN: 1.35 ± 0.56 TbTh: 0.071 ± 0.020 *Distal radius* BV/TV: 7.39 ± 3.16 TbN: 1.36 ± 0.46 TbTh: 0.055 ± 0.017	12 months *Distal tibia* BV/TV: 8.66 ± 3.36 TbN: 1.33 ± 0.49 TbTh: 0.067 ± 0.018 *Distal radius* BV/TV: 7.14 ± 3.03 TbN: 1.34 ± 0.41 TbTh: 0.054 ± 0.017	*Distal tibia* BV/TV: 8.70 ± 3.06 TbN:1.34 ± 0.45 TbTh: 0.066 ± 0.013 *Distal radius* BV/TV: 7.63 ± 3.34 TbN: 1.33 ± 0.54 TbTh: 0.059 ± 0.018	*6 months* *Distal tibia* BV/TV: 8.82 ± 3.47 TbN: 1.32 ± 0.49 TbTh: 0.068 ± 0.015 *Distal radius* BV/TV: 7.93 ± 3.52 TbN: 1.34 ± 0.51 TbTh: 0.060 ± 0.018	12 months *Distal tibia* BV/TV: 8.70 ± 3.34 TbN: 1.36 ± 0.48 TbTh: 0.065 ± 0.015 Distal radius BV/TV: 7.91 ± 3.69 TbN: 1.41 ± 0.48 TbTh: 0.055 ± 0.013
Liphardt, 2015 [46]	XtremeCT, Scanco Medical	Non-dominant radius and left tibia. Participants who reported a previous fracture of the radius or tibia were scanned at the dominant limb	*Tibia* BV/TV: 12.6 ± 2.2 TbN: 1.69 ± 0.23 TbTh: 0.075 ± 0.012 TbSp: 0.526 ± 0.083 *Radius* BV/TV: 10.2 ± 3.0 TbN: 1.72 ± 0.35 TbTh: 0.059 ± 0.010 TbSp: 0.557 ± 0.199	4 months *Tibia* BV/TV: 12.5 ± 2.2 TbN: 1.63 ± 0.23 TbTh: 0.077 ± 0.011 TbSp: 0.548 ± 0.081 *Radius* BV/TV: 10.2 ± 3.0 TbN: 1.74 ± 0.35 TbTh: 0.058 ± 0.008 TbSp: 0.550 ± 0.189 8 months *Tibia* BV/TV: 12.5 ± 2.2 TbN: 1.72 ± 0.23 TbTh: 0.073 ± 0.008 TbSp: 0.519 ± 0.080 *Radius* BV/TV: 10.2 ± 3.1 TbN: 1.73 ± 0.38 TbTh: 0.059 ± 0.010 TbSp: 0.560 ± 0.209	12 months *Tibia* BV/TV: 12.5 ± 2.1 TbN: 1.74 ± 0.28 TbTh: 0.073 ± 0.012 TbSp: 0.517 ± 0.096 *Radius* BV/TV: 10.2 ± 3.1 TbN: 1.72 ± 0.36 TbTh: 0.059 ± 0.009 TbSp: 0.563 ± 0.214	*Tibia* BV/TV: 12.0 ± 3.2 TbN: 1.53 ± 0.25 TbTh: 0.078 ± 0.015 TbSp: 0.592 ± 0.112 *Radius* BV/TV: 11.0 ± 3.9 TbN: 1.83 ± 0.33 TbTh: 0.059 ± 0.014 TbSp: 0.507 ± 0.119	4 months *Tibia* BV/TV: 12.1 ± 3.1 TbN: 1.59 ± 0.26 TbTh: 0.075 ± 0.012 TbSp: 0.570 ± 0.125 *Radius* BV/TV: 10.9 ± 4.0 TbN: 1.80 ± 0.37 TbTh: 0.059 ± 0.013 TbSp: 0.520 ± 0.136 8 months *Tibia* BV/TV: 11.9 ± 3.2 TbN: 1.53 ± 0.28 TbTh: 0.078 ± 0.014 TbSp: 0.598 ± 0.132 *Radius* BV/TV: 10.9 ± 4.0 TbN: 1.79 ± 0.35 TbTh: 0.059 ± 0.013 TbSp: 0.520 ± 0.126	12 months *Tibia* BV/TV: 12.0 ± 3.1 TbN: 1.56 ± 0.27 TbTh: 0.077 ± 0.015 TbSp: 0.581 ± 0.119 *Radius* BV/TV: 10.9 ± 3.9 TbN: 1.77 ± 0.31 TbTh: 0.061 ± 0.016 TbSp: 0.523 ± 0.122
Ng, 2021 [47]	XtremeCT II, Scanco Medical	Non-dominant tibia	BV/TV: 20.4 ± 4.3* TbN: 1.150 ± 0.301 TbTh: 0.247 ± 0.018 TbSp: 0.944 ± 0.424	N/A	16 weeks BV/TV: 20.4 ± 4.3* TbN: 1.159 ± 0.307 TbTh: 0.248 ± 0.019 TbSp: 0.947 ± 0.446	N/A	N/A	N/A
Pinho, 2020 [48]	XtremeCT, Scanco Medical	Dominant tibia	Mean (95% CI) BV/TV: 12.0 (10.5–13.5)* TbN: 1.684 (1.547–1.821) TbTh: 0.071 (0.065–0.077) TbSp: 0.543 (0.482–0.604)	N/A	20 weeks Mean (95% CI) BV/TV: 12.0 (10.4–13.6)* TbN: 1.620 (1.464–1.776) TbTh: 0.073 (0.067–0.079) TbSp: 0.577 (0.502–0.652)	Mean (95% CI) BV/TV: 10.8 (9.3–12.3)* TbN: 1.638 (1.473–1.803) TbTh: 0.066 (0.059–0.073) TbSp: 0.572 (0.491–0.653)	N/A	20 weeks Mean (95% CI) BV/TV: 10.5 (9.0–12.0)* TbN: 1.653 (1.478–1.828) TbTh: 0.063 (0.056–0.070) TbSp: 0.574 (0.482–0.666)
Sundh, 2018 [49]	XtremeCT, Scanco Medical	Tibia of both legs	BV/TV: 11.7 ± 2.8 TbN: 1.73 ± 0.25 TbTh: 0.0676 ± 0.0113**	N/A	3 months BV/TV: 11.8 ± 2.8 TbN: 1.70 ± 0.25 TbTh: 0.0689 ± 0.0111**	BV/TV: 11.5 ± 2.5 TbN: 1.71 ± 0.25 TbTh: 0.0671 ± 0.0115**	N/A	3 months BV/TV: 11.4 ± 2.6 TbN: 1.70 ± 0.23 TbTh: 0.0668 ± 0.0123**
Slatkovska, 2011 [50]	XtremeCT, Scanco Medical	Distal tibia and distal radius (limb not specified)	90-Hz WBV *Distal tibia* BV/TV: 12.4 ± 3.0* TbN: 1.56 ± 0.25 TbTh: 0.079 ± 0.016 TbSp: 0.578 ± 0.118 *Distal radius* BV/TV: 11.4 ± 2.9* TbN: 1.73 ± 0.26 TbTh: 0.066 ± 0.011 TbSp: 0.526 ± 0.095 30-Hz WBV *Distal tibia* BV/TV: 12.0 ± 2.4* TbN: 1.50 ± 0.28 TbTh: 0.081 ± 0.013 TbSp: 0.610 ± 0.127 *Distal radius* BV/TV: 11.5 ± 2.4* TbN: 1.71 ± 0.27 TbTh: 0.067 ± 0.010 TbSp: 0.530 ± 0.093	N/A	Mean absolute change at 12 months (95% CI) 90-Hz WBV *Distal tibia* BV/TV: 0.0 (0.0 to 0.1)* TbN: 0.07 (0.03 to 0.10) TbTh: -0.003 (-0.005 to -0.002) TbSp: -0.022 (-0.033 to -0.011) *Distal radius* BV/TV: -0.2 (-0.3 to 0.0)* TbN: 0.03 (-0.01 to 0.07) TbTh: -0.002 (-0.003 to 0.000) TbSp: -0.002 (-0.018 to 0.015) 30-Hz WBV *Distal tibia* BV/TV: 0.0 (-0.1 to 0.1)* TbN: 0.08 (0.04 to 0.11) TbTh: -0.004 (-0.005 to -0.002) TbSp: -0.023 (-0.034 to -0.011) *Distal radius* BV/TV: -0.1 (-0.3 to 0.0)* TbN: 0.03 (-0.01 to 0.08) TbTh: -0.002 (-0.004 to -0.001) TbSp: -0.009 (-0.026 to 0.008)	*Distal tibia* BV/TV: 12.0 ± 2.5* TbN: 1.54 ± 0.27 TbTh: 0.079 ± 0.015 TbSp: 0.592 ± 0.125 *Distal radius* BV/TV: 11.6 ± 2.7* TbN: 1.73 ± 0.28 TbTh: 0.067 ± 0.012 TbSp: 0.530 ± 0.130	N/A	Mean absolute change at 12 months (95% CI) *Distal tibia* BV/TV: 0.0 (-0.1 to 0.0)* TbN: 0.06 (0.02 to 0.09) TbTh: -0.002 (-0.004 to -0.001) TbSp: -0.018 (-0.030 to -0.006) *Distal radius* BV/TV: -0.1 (-0.2 to 0.0)* TbN: 0.02 (-0.02 to 0.07) TbTh: -0.001 (-0.003 to 0.000) TbSp: -0.008 (-0.025 to 0.009)

*HR-pQCT* high-resolution peripheral quantitative computed tomography, *BV/TV* trabecular bone volume fraction, *TbN* trabecular number, *TbTh* trabecular thickness, *TbSp* trabecular separation, *CI* confidence interval, *WBV* whole-body vibration. Units: trabecular bone volume fraction = percentage, trabecular thickness = millimetres, trabecular separation = millimetres, trabecular number = per millimetre. All parameters are denoted as mean ± standard deviation unless otherwise stated. *Converted from decimal to percentage; **Converted from micrometres to millimetres.

### Meta-analysis

**Fig. 2:**
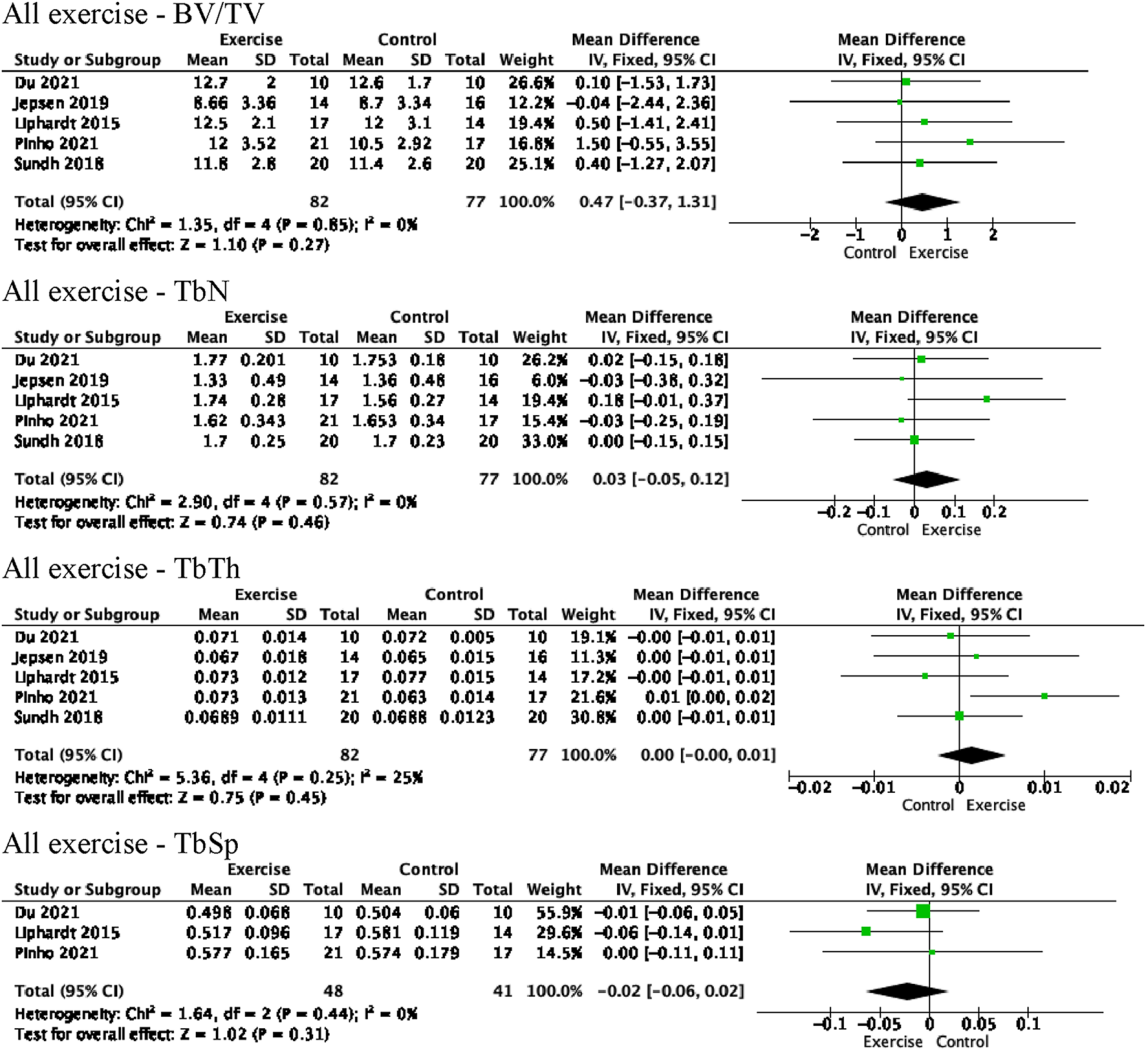
Meta-analysis of the effect of all exercise interventions on trabecular bone volume fraction (BV/TV), trabecular number (TbN), trabecular thickness (TbTh), and trabecular separation (TbSp) in the distal tibia. *SD* standard deviation, *IV* inverse variance, *CI* confidence interval

Two studies were not included in the meta-analysis due to their lack of control group [47] and follow-up data [50]. Meta-analysis of the remaining five studies suggests that exercise did not have significant effects on any TbM parameters at either the tibia or radius (Fig. 2, Online Resources 2 and 3). Heterogeneity was low for all parameters when analysing all exercise interventions and sub-analysing based on type of exercise, increasing confidence that the interventions had minimal effect on TbM. The power and plyometric exercise programme was not sub-analysed because there was only one study [48].

Percent change of BV/TV, TbN, TbTh, and TbSp between baseline and follow-up measurements in the intervention groups are depicted graphically in Online Resources 4 and 5 for the tibia and radius, respectively. No studies reported significant changes to any radial TbM parameters. In the tibia, no significant changes in BV/TV were reported. Jepson et al. [45], however, observed a different trend than all other studies, noting a continuously decreasing BV/TV in both the tibia and radius between baseline and 12 months (Online Resources 4a and 5a).

Two studies [44, 46] found statistically significant increases in tibial TbN. Du et al. [44] observed an increase in global TbN when measuring after their six-month intervention (p < 0.05, leg and time interaction). Liphardt et al. [46] noted an initial decrease in the first four months of their WBV intervention, followed by a greater increase for the next eight months (p = 0.043, time and group interaction). Overall, a general trend towards increased tibial TbN with exercise over time can be observed in the jumping and WBV groups, apart from Jepson et al. [45] (Online Resource 4b). This trend was not observed in the radius (Online Resource 5b).

Two studies [46, 48] reported significant changes in tibial TbTh. Liphardt et al. [46] noted an initial increase at their four-month tibial measurements, followed by a greater decrease at month eight, which remained at one year. It must be noted that a p = 0.05 was reported in text, however, a table revealed the true p-value to be 0.052 (time and group interaction), indicating that, although close, the result was not statistically significant. Pinho et al. [48] also observed a significant increase in tibial TbTh when assessing after their 20-week intervention (p = 0.027). This was the only parameter in the meta-analysis that did not cross the line of null effect (Fig. 2). A trend towards decreased tibial TbTh over time after an initial rise can be observed in the WBV studies, while no obvious trend can be observed for the jumping studies or when considering all exercise interventions together (Online Resource 4c). In the radius, a statistically insignificant trend towards decreased TbTh with exercise over time can be observed, although Liphardt et al. [46] deviate from this pattern (Online Resource 5c).

Finally, Liphardt et al. [46] was the only study to note statistically significant changes in tibial TbSp, observing an initial increase at their four-month measurement followed by greater decreases for the subsequent eight months (p = 0.047, time and group interaction). Overall, a trend towards decreased TbSp with WBV over time can be seen in the tibia (Online Resource 4d). As TbSp was only examined in two jumping studies, it is difficult to extrapolate any trends. No tendencies can be seen in the radius (Online Resource 5d).

### Quality Appraisal

Results of the EPHPP quality assessment can be found in Online Resource 6. Six studies [44–46, 48–50] received a global rating of moderate, and one [47] received a global rating of weak. Based on the global ratings of these studies, credibility was assigned to their findings.

## Discussion

This systematic review and meta-analysis examined the effects of exercise on HR-pQCT derived TbM parameters in adults aged over 50. Overall, exercise did not have a significant effect on BV/TV, TbN, TbTh, or TbSp. Based on the quality assessment results, the findings of this review should be interpreted with caution.

To our knowledge, this is the first review examining the effects of exercise on TbM in older adults. The impact of exercise on other trabecular bone parameters, however, has been investigated in postmenopausal women, with inconsistent conclusions. Findings of Hamilton et al. [37] were not definitive—only four of their eight included studies that examined trabecular BMD and content showed evidence of exercise-induced adaptations. Only two were controlled trials, and they had differing results. Although they included cross-sectional and prospective cohort studies that showed positive associations between exercise and trabecular bone, these cannot be used to establish causality [53]. Subsequently, Polidoulis et al. [38] found that exercise had similar effects on cortical and trabecular volumetric BMD. While their review was more robust with a broader search strategy, completion of a meta-analysis, and inclusion of only RCTs, four of their six included studies were also evaluated by Hamilton et al. [37]. Both reviews therefore face similar hindrances. The small number of RCTs and inclusion of only postmenopausal women limits the generalisability of their results. These limitations also apply to the current review. While the findings of this review align more with those of Hamilton et al. [37], the scarcity of studies must be considered when inferring an absence of exercise-induced effects. Moreover, because of the possible differences in exercise-induced changes in TbM compared to BMD and content, these findings cannot be directly compared.

As all studies in this review were in postmenopausal women, these findings are not representative of the original target population of all adults aged 50 and over. Furthermore, because most of the studies took place in countries with large white populations, results may not be generalisable to other races and ethnicities. Based on the differences in BMD, fragility fracture risk, and osteoporosis incidence between racial and ethnic groups [54–56], it is sensible to think that TbM may also differ. Additionally, five studies excluded individuals with osteoporosis, a group for whom enhancing bone strength is especially important. As such, the generalisability of this review’s findings is limited to the investigated subgroup of postmenopausal women.

Only three types of exercise were utilised by studies in this review, further limiting the generalisations that can be made. Although negligible changes in TbM were observed after single-leg jumping interventions, it must be noted that the longest was six months, which is the minimal time required to see exercise-induced changes in BMD [57]. As such, these studies may have been too short to elicit TbM adaptations. Longer duration RCTs are needed to determine how TbM changes over time in response to impact exercise. Nonetheless, impact activities are beneficial to bone health in postmenopausal women, especially when performed alongside resistance training [58]. Performance of impact activities on a regular basis is recommended for adults over 65 who can perform them safely [5, 59]. Even in those with osteoporosis, up to moderate-level impact activities are recommended for those free of vertebral fractures or more than one low-trauma fracture [59]. These guidelines should be adhered to until research suggests otherwise.

The only significant change in TbM resulting from jumping interventions was the increased TbN after six months reported by Du et al. [44]. However, their trial included only 10 participants from a larger study [51] who volunteered to have HR-pQCT scans taken. This small sample size and lack of explanation as to why this number was chosen impairs the estimate of random error [60]. Moreover, participants volunteering for additional scans may be more health-conscious than the general population, increasing the risk of selection bias. Lastly, while randomising the intervention leg within participants helped minimise baseline differences, systemic effects of exercise could also have impacted the control limb [44].

Pinho et al. [48] was the only study to use a combined power and plyometric exercise intervention. Interestingly, they reported an increase in TbTh after 20 weeks. Six months are generally required before exercise-induced changes in BMD can be detected [57]—these findings suggest that the initiation of exercise-induced TbM adaptations may occur before those in BMD. As metabolic activity is greater in trabecular than cortical bone [25], one could reasonably think it would begin to adapt first. This study was appropriately powered and had no dropouts, high adherence, and provided a detailed explanation of their intervention, which aids future researchers looking to conduct similar studies. More RCTs measuring TbM parameters in response to exercise at various timepoints throughout the intervention are required to determine when adaptations begin.

The absence of noted WBV-induced TbM changes in this review parallel findings of a systematic review by Oliveira et al. [61], which did not find significant changes in trabecular volumetric BMD at the tibia or radius. However, their review only included three and four studies examining trabecular volumetric BMD at the radius and tibia, respectively. Moreover, two of these studies are included in the current review, demonstrating the scarcity of research on the effects of WBV on trabecular bone at these regions. A limitation of both reviews is the heterogeneity of WBV protocol, which hinders comparison between studies [61]. Nonetheless, as lumbar spine BMD adaptations elicited by WBV are promising [52, 61], in addition to its feasibility for older adults who struggle with more intensive types of exercise, continuing to investigate which combination of parameters elicits the greatest adaptations in bone strength is important.

Liphardt et al. [46] were the only WBV study to report significant changes to TbM, with increased TbN and TbTh alongside decreased TbSp. Akin to the other studies with statistically significant results, selection bias was an issue. As sessions were supervised in a clinic multiple times per week for a year, participants were likely more motivated to adhere than the general population [46]. Furthermore, participants in the other WBV studies were either osteoporotic and taking medication [45] or were osteopenic but met extensive eligibility criteria and received calcium and vitamin D supplementation [50]. Participants in this study were simply osteopenic. Each intervention also used different machine parameters, which may have had differing osteogenic effects. These differences between participant characteristics and interventions could have contributed to the varying results. It is also important to note that the minimal clinically important difference for TbM parameters have not been established, therefore the extent to which statistically significant changes are clinically relevant is unknown.

Although their results were not statistically significant, Jepsen et al. [45] showed the greatest percentage decrease of all studies in every parameter they measured except for tibial TbTh. As they were the only study to administer teriparatide treatment to participants, one explanation is that the deleterious effects on bone quality were due to an interaction between the medication and WBV training. Despite being an established anabolic therapy for osteoporosis, two studies [62, 63] have found inconsistent effects of teriparatide treatment on TbM at the distal tibia and radius. As neither adjusted for the possible effects of exercise or PA, these factors could have interacted with the effects of the medication and impacted results. Jepson et al. [45] also had limitations in their study that may have contributed to their findings. The lack of WBV supervision and self-administration of teriparatide are realistic with regards to how these interventions would be used as treatments, however participants may not have been completing them correctly. In addition, power calculations were based on lumbar spine BMD, therefore the study was likely to be underpowered to detect changes in TbM [45]. Regardless of these limitations, the outcomes of this study are important because the combined use of medication and exercise is a realistic treatment for osteoporosis, and it is important to ensure that treatments do not harm patients. As the first study to examine their combined effects on TbM, it provides a framework for future RCTs to explore similar interventions.

On examination of the graphs in Online Resources 4 and 5, various trends can be observed between TbM parameters in the intervention groups. At both the tibia and radius, changes in BV/TV were minimal and did not strongly correlate with changes to any other parameters. TbN tended to have an inverse relationship with TbTh and TbSp, while the latter two generally had a positive relationship with each other. This may be due to thinner trabeculae being resorbed while those remaining become thicker, resulting in minimal change in BV/TV [29, 48]. While these relationships between parameters are similar to those seen during the age-related degradation of TbM [26], the maintenance of BV/TV suggests the preservation of trabecular bone [48]. These general trends were similar in the tibia and radius, however less pronounced in the latter. Although the radius was only scanned in the WBV studies, the less prominent effects are consistent with bone’s site-specific adaptations to stress [64]. Based on this localised response, it is also possible that the distal sites of the tibia and radius that were measured were not adequately loaded to stimulate adaptation, while other unmeasured regions were. It would have been interesting to see TbM measurements of the distal radius in the study by Pinho et al. [48] because their exercise programme included two upper body exercises.

As age-related changes to TbM parameters differ between men and women [26], research in older men is required to determine whether there are differences in exercise-induced changes between sexes in older adults. Hughes et al. [65] found that in young adults undergoing intensive periods of physical activity during military training, improvements in TbM were seen in men and women, but women had greater increases BV/TV and TbTh. Studies examining differences in exercise-induced adaptations between older men and older women are required to see if these variations differ throughout the lifespan.

The individual and societal consequences of fragility fractures are recognised worldwide [66]. Studies in this review were recently published and took place in six countries across four continents, making it apparent that strategies to minimise these consequences are sought after globally. Bone quality presents an unexplored avenue that may play a role in diminishing these ramifications.

### Strengths and Limitations

Limitations must be considered when interpreting these results. Further refinement may have improved the search strategy, evidenced by the discovery of an eligible study while citation searching when only six were found via the database search. Including a more comprehensive set of search terms, such as terms for specific types of exercise, would have helped to minimise the risk of missing relevant studies. Moreover, utilising more key databases in addition to contacting authors and experts in the field to enquire about other studies would have also increased the rigor of this review. Inclusion of only English publications may have excluded studies which would have otherwise satisfied the eligibility criteria. By only including studies that used HR-pQCT as the measurement tool, otherwise relevant studies may have also been excluded. Additionally, as HR-pQCT can only scan the distal limbs, changes in TbM at other important sites like the hip and spine could not be detected. Comparison between the XTremeCT I and II may have been inappropriate due to improvements in the second-generation model [34], albeit only one study [47] used the second-generation device. In addition, as the skeletal site loaded differs based on the type of exercise, incorporating multiple types may have increased the heterogeneity and reduced the likelihood of detecting an overall effect. Limitations of this review also extend to the included studies. The low number of studies and small sample size further limit the generalisations that can be made. Their weak to moderate quality and high risk of selection bias indicate that findings must be interpreted with caution. Lastly, as TbM is only one element of bone quality, these findings are not representative of any other components or of bone quality overall.

This review also has several strengths. It is the first to examine the effects of exercise on TbM, therefore contributing to an emerging area of study. Having a second independent reviewer during the literature screening and data extraction processes helped minimise the risk of mistakes. Finally, the inclusion of studies which used HR-pQCT as their bone imaging device facilitated comparison between studies and ensured that results were accurate and standardised.

### Future Research and Practical Applications

The findings of this review do not suggest that any changes to professional practice or exercise recommendations should be made. Older adults should continue to follow current guidelines to optimise bone health and minimise fracture risk.

As mentioned, there is a need for further research. RCTs longer than six months examining exercise-induced changes in TbM, taking measurements at multiple timepoints throughout the intervention period, should be conducted for various types of exercise. These additional criteria would help determine when TbM adaptations begin to occur, how they manifest over time, and how they differ based on mode of exercise. The interventions examined in this review may have feasibility, safety, and in the case of WBV, financial barriers for older adults. Future RCTs should therefore include types of exercise that are practical for an older population and do not have monetary implications. Due to the homogeneity of the participants in this review, further research is required in more diverse populations of adults over 50, including males and individuals of differing races and ethnicities. Lastly, as TbM is only one aspect of bone quality, future systematic reviews should investigate the effects of exercise on other components.

## Conclusion

This review found no significant exercise-induced changes to any TbM parameters in postmenopausal women. These conclusions must be taken with caution due to the small number of studies, their weak to moderate quality, high risk of selection bias, and the few types of exercise investigated. Further research incorporating additional types of exercise in a diverse range of older adults is required. Regardless of these findings, exercise is important for older adults to preserve bone health and improve quality of life.

### Supplementary Information

Below is the link to the electronic supplementary material.

Supplementary file1 (DOCX 4452 kb)

## Data Availability

Extracted data for the review is available from the authors.
