# Supplementary material for: What are the Effects of Exercise on Trabecular Microarchitecture in Older Adults? A Systematic Review and Meta-analysis of HR-pQCT Studies
Source: Calcif Tissue Int. 2023 Sep 19;113(4):359–82. doi: 10.1007/s00223-023-01127-7 (PMC10516781; doi:10.1007/s00223-023-01127-7)
Supplement: Supplementary file 1 — Supplementary file1 (DOCX 4452 kb) [file 223_2023_1127_MOESM1_ESM.docx]

**Online Resources -** What are the effects of exercise on trabecular microarchitecture in older adults? A systematic review and meta-analysis of HR-PQCT studies.

**Calcified Tissue International**

**Authors:** Thomas Z Schlacht, Inaya Haque, Dawn A Skelton

**Institution:** Glasgow Caledonian University, Cowcaddens Road, Glasgow, G4 0BA, UK.

**Department:** Research Centre for Health (ReaCH), Physiotherapy and Paramedicine

**Correspondence:** Dawn A Skelton; dawn.skelton@gcu.ac.uk

**Online Resource 1**: Search strategy

The following terms were used to create the final search for each database. Boolean operator “OR” was used to combine phrases within categories, and “AND” was used to combine categories. Relevant Subject Headings and Medical Subject Headings were also used as able in each database.

**Population**

Adult*

OR

“Older adult*”

OR

Ag#ing

OR

Aged

OR

“Middle age*”

OR

Elderly

OR

Geriatric

**Intervention**

Exercis*

OR

"Physical activity”

**Outcome**

“Bone quality”

OR

“Bone geometry”

OR

“Bone structure”

OR

“Bone strength”

OR

“bone micro*”

OR

“trabecular micro*”

OR

Trabecula*

OR

“trabecular bone”

OR

“Cancellous bone”

OR

“Peripheral quantitative computed tomography”

OR

PQCT

OR

“high resolution peripheral quantitative computed tomography”

OR

“HR PQCT”

**Online Resource 2:** Meta-analysis of the effect jumping and whole-body vibration (WBV) exercise interventions on trabecular bone volume fraction (BV/TV), trabecular number (TbN), and trabecular thickness (TbTh) in the distal tibia.

Abbreviations: SD = standard deviation, IV = inverse variance, CI = confidence interval.

Jumping - BV/TV


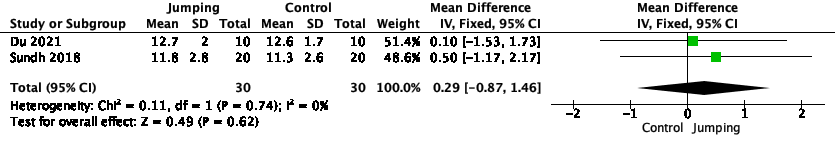


WBV - BV/TV


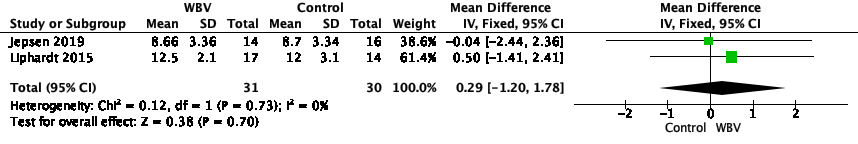


Jumping - TbN


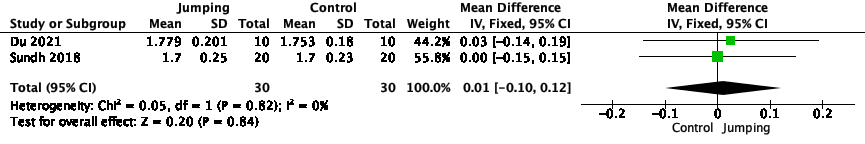


WBV - TbN


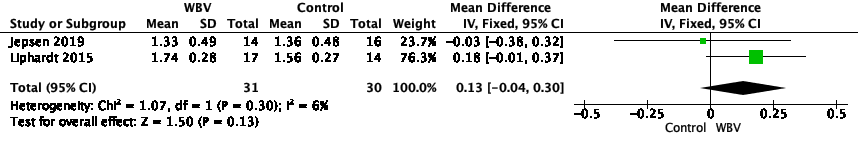


Jumping - TbTh


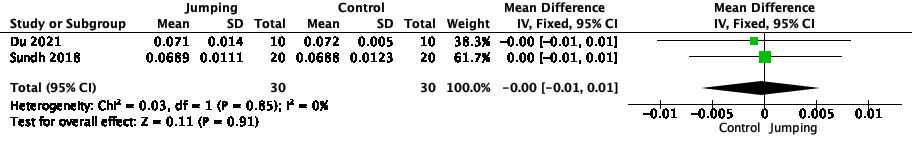


WBV - TbTh


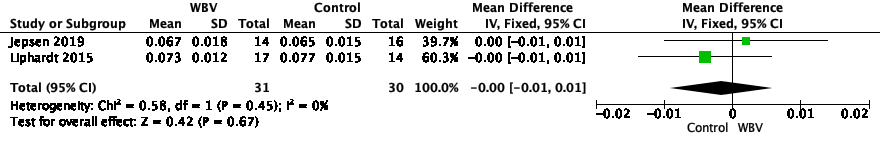


**Online Resource 3:** Meta-analysis of the effect of whole-body vibration (WBV) exercise on trabecular bone volume fraction (BV/TV), trabecular number (TbN), and trabecular thickness (TbTh) in the distal radius.

Abbreviations: SD = standard deviation, IV = inverse variance, CI = confidence interval.

WBV - BV/TV


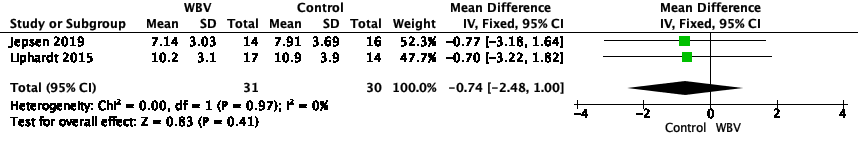


WBV - TbN


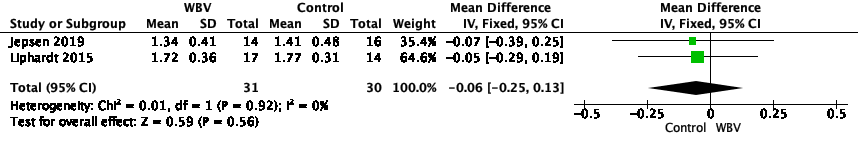


WBV - TbTh


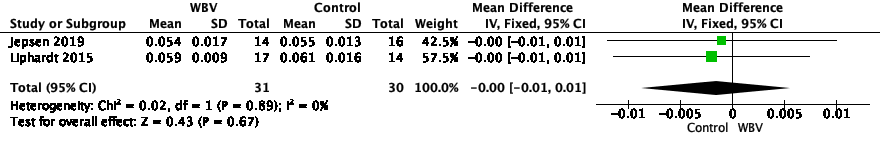


**Online Resource 4:** Percentage change in tibial (a) trabecular bone volume fraction (BV/TV), (b) trabecular number (TbN), (c) trabecular thickness (TbTh), and (d) trabecular separation (TbSp) between pre- and post-intervention measurements. In cases where studies took measurements during the intervention period, intermediate and post-intervention data points are connected. Results reported in weeks were converted to months under the assumption that four weeks equated to one month.

**Online Resource 5:** Percentage of change in radial (a) trabecular bone volume fraction (BV/TV), (b) trabecular number (TbN), (c) trabecular thickness (TbTh), and (d) trabecular separation (TbSp) between pre- and post-intervention measurements. In cases where studies took measurements during the intervention period, intermediate and post-intervention data points are connected.

**Online Resource 6:** Results of quality assessment using EPHPP Quality Assessment Tool for Quantitative Studies.

| **Article** | **Selection bias** | **Study design** | **Confounders** | **Blinding** | **Data collection methods** | **Withdrawals and dropouts** | **Global rating** |
| --- | --- | --- | --- | --- | --- | --- | --- |
| Du, 2021 [44] | Weak | Strong | Strong | Moderate | Strong | Strong | Moderate |
| Jepsen, 2019 [45] | Weak | Strong | Strong | Moderate | Strong | Strong | Moderate |
| Liphardt, 2015 [46] | Weak | Strong | Strong | Moderate | Strong | Moderate | Moderate |
| Ng, 2021 [47] | Weak | Moderate | Weak | Moderate | Strong | Strong | Weak |
| Pinho, 2020 [48] | Weak | Strong | Strong | Moderate | Strong | Strong | Moderate |
| Sundh, 2018 [49] | Weak | Strong | Strong | Moderate | Strong | Strong | Moderate |
| Slatkovska, 2011 [50] | Weak | Strong | Strong | Moderate | Strong | Strong | Moderate |
